# Robusta Coffee Husk Extract Increases the Number of Fibroblast and Collagen Density in Gingival Rat Periodontitis

**DOI:** 10.1155/sci5/8952540

**Published:** 2025-04-18

**Authors:** Nadie Fatimatuzzahro, Rendra C. Prasetya, Amandia D. P. Shita, Nuzulul Hikmah, Hafiedz Maulana, Dwi K. Apriyono, Kenanga D. Prameiswari, Hanna A. Pratiwi

**Affiliations:** ^1^Department of Dentistry, Biomedical Science, Oral Pathology and Maxillofacial, Faculty of Dentistry, Universitas Jember, Jember 68121, Indonesia; ^2^Department of Dentistry, Forensic Odontology, Faculty of Dentistry, Universitas Jember, Jember 68121, Indonesia; ^3^Department of Dentistry, Faculty of Dentistry, Universitas Jember, Jember 68121, Indonesia

**Keywords:** antibacterial, anti-inflamatory, antioxidant, *Coffea robusta* husk, *Phorphyromonas gingiva*lis

## Abstract

**Introduction:** Periodontitis is an infectious disease of periodontal tissue caused by the bacteria *Porphyromonas gingivalis* which can trigger the formation of free radicals. Periodontitis can be treated using metronidazole but long-term use can cause negative effects. Natural ingredients such as robusta coffee husk can be used as an alternative treatment for periodontitis because of its anti-inflammatory, antioxidant, and antibacterial benefits.

**Aim:** To determine the effect of robusta coffee husk extract on the number of fibroblasts and collagen density in gingival Wistar rats induced by *P. gingivalis*.

**Methods:** Robusta coffee husk extract was extracted using the maceration process with 96% ethanol. Thirty male Wistar rats divided into 6 treatment groups: healthy rat, negative control (*P. gingivalis* + Aquades sterile), positive control (*P. gingivalis* + metronidazole), and 3 treatment groups of extract concentration (*P. gingivalis* + 125, 250, and 500 mg/kgBW). *P. gingivalis* injection was carried out in the buccal area of the left mandibular first molar of mice every 3 days. Robusta coffee husk extract and metronidazole were given by using sondase once a day for 21 days. The gingival specimens were then processed histologically. Hematoxylin and Eosin (H&E) staining was performed to observe the number of fibroblast, and collagen density was observed by Thricome Mallory. The SPSS application was used to examine the data calculation which included the Shapiro–Wilk, Levene, One-way ANOVA, and post hoc LSD tests.

**Result:** Robusta coffee husk extract at doses of 125, 250, and 500 mg/kgBW showed increased fibroblasts and collagen density in Wistar rats induced by *P. gingivalis.*

**Conclusion:** The most effective dose of robusta coffee husk extract is 250 mg/kgBW, with an average value of fibroblast number in a field of view is 37 cells, and the average of collagen density in the field of view is 163 pixels.

## 1. Introduction

Periodontitis is a disease that occurs in the oral cavity and affects almost 90% of the global population [1]. Periodontitis causes pathological conditions in the gingiva, periodontal ligament, and alveolar bone. The most common etiology of periodontitis is the pathogenic bacteria *Porphyromonas gingivalis* [2]. *P. gingivalis* is an anaerobic Gram-negative bacterium that colonizes oral tissue, especially subgingival plaque [3].

*P. gingivalis* has virulence factors including capsule, fimbriae, lipopolysaccharide, and gingipain, which can activate the immune response [3]. One of the cells that play a role in the immune response is macrophages. Macrophages produce a large group of proinflammatory cytokines such as interleukin 1β (IL-1β), interleukin 6 (IL-6), tumor necrosis factor alpha (TNF-α), and matrix metalloproteinase (MMP) enzymes. Increased MMP enzymes can cause excessive collagen degradation in soft tissue [2, 4, 5].

Periodontitis can be treated with curettage, scaling and root planning, and administering antibiotics such as metronidazole. Based on the research results of Pang et al., a dose of metronidazole 200 mg/kgBW is effective as periodontitis therapy in mice. Long-term use of chemical-based metronidazole can cause side effects such as local irritation and allergies; therefore, other alternative ingredients are needed that are safer and do not cause side effects [6]. One natural ingredient that can be used is robusta coffee husk. Secondary compounds such as flavonoids and phenols contained in the husk of the coffee robusta can be useful as anti-inflammatory, antioxidant, and antibacterial [7–9].

The periodontitis healing process consists of 3 phases including the inflammatory phase, proliferation phase, and remodeling phase. One of the cells that has an important role in the proliferation phase is fibroblasts. Fibroblasts are one of the cells found in connective tissue and will migrate towards the inflammatory area to stimulate collagen formation gradually [10]. The number of fibroblasts will increase starting from Day 3 and will continue to increase until Day 21, and the maturation process of collagen fibers will also occur [11].

This study has proven the potential of robusta coffee husk extract as a candidate for periodontitis therapy. Robusta coffee husk is a waste product of the coffee production process contains flavonoid compounds and phenols that can be useful as anti-inflammatory, antioxidant, and antibacterial [7, 9]. Previous studies demonstrated that robusta coffee husk extract can suppress the growth of *P. gingivalis* and *E. faecalis* [12]. However, no research has been conducted on the effect of robusta coffee husk extract at a dose of 125, 250, and 500 mg/kgBW on the number of fibroblasts and collagen density in Wistar rats induced by *P. gingivalis* for 21 days.

## 2. Materials and Methods

Ethical approval for this research (no. 2239/UN25.8/KEPK/DL2023) was approved by the Health Research Ethics Committee, Faculty of Dentistry, Jember University. This is a laboratory experimental research, with the posttest only control group design. Thirty Wistar rats with an average body weight of 200–250 g, healthy, and had no abnormalities were divided into 6 groups: normal group, positive control (metronidazole 200 mg/kgBW), negative control (sterile distilled water), and robusta coffee husk extract at doses of 125 250, and 500 mg/kgBW. Robusta coffee husk extract and metronidazole were given by using sondase once a day for 21 days.

Wistar rats were chosen for research on periodontitis because these rats have similar characteristics to humans in terms of dental anatomy, immune system, and inflammatory response in periodontitis. Apart from that, Wistar rats are also easy to care for and have a body size that is large enough to make sampling easier [13].

• Tools: blender (Philips Holland), oven (Binder, Germany), Erlenmeyer flask (Schott Duran, Germany), digital scale (Ohaus, USA), rotary evaporator (Heidolph, Germany), maceration jar (No Brand), filter paper (Cytiva, China), spatula (No Brand), 1.5 mL Eppendorf tube (No Brand), 50 mL falcon tube (Onemed, Indonesia), gastric sonde (Obsidi Medica, Indonesia), mask (Onemed, Indonesia), gloves (Onemed, Indonesia), plastic filling instrument (Schezher, Germany), tweezers (Onemed, Indonesia), Petri dish (Iwaki Pyrex, Japan), loop needle (Nikrom, Indonesia), micropipette (Dragon Lab, China), incubator (Binder, Germany), vortex (Faithful, China), microtome (Tisuue-Tek, Japan), glass slide (Sail Brand, China), glass slide cover (Menzel Glaser, Germany), water bath (Memmert, Germany), small brush (Lyra, Taiwan), electric stove (Maspion, Indonesia), light microscope (Olympus, Japan), Optilab (Obtilab Advance, Indonesia), and autoclave (GEA, China).
• Materials: thirty male Wistar rats, standard litter (Turbo, Indonesia), drinking water (Aqua, Indonesia), robusta coffee husk (No Brand), sterile distilled water (USFA, Indonesia), *P. gingivalis* suspension (MediMark, France), TSA bacterial culture material (Thryptone Soya Agar), vitamin K (Sigma, Germany), hemin (MP Biomedicals Inc, France), paraffin (Sigma-Aldrich, Germany), Hematoxylin and Eosin (H&E) (Leica, Netherlands), Mallory Trichrome (ScyTek, USA), ethanol 96% (Supelco, Germany), metronidazole (Indonesia), ketamine (KTM-100, Indonesia), alcohol 70% (Onemed, Indonesia), xylol (Supelco, Germany), buffered formalin, physiological NaCl 0.9%, tissue (Paseo, Indonesia), cotton palette (No Brand), 5 mL and 1 mL disposable syringe (Onemed, Indonesia), and 30G needle (MediMark, France).

### 2.1. Preparation of Robusta Coffee Husk Extract

Robusta coffee husk extract begins by soaking 800 g of robusta coffee husk powder in a maceration bottle for 3 days with the addition of 96% ethanol solvent using a ratio of 1:5 and stirring once a day and then filtered using fine filter paper and evaporated using a temperature of 60°C for 12 h in a rotary evaporator.

### 2.2. Preparation Concentration of Robusta Coffee Husk Extract

The single dose was given according to the initial weight of the rat. Preparing the extract dose was carried out by placing robusta coffee husk extract into a test tube containing 1 mL of sterile distilled water and mixing until homogeneous using a vortex. This research was carried out with dose selection based on the research conducted by Cahyani, which states that a dose of 125, 250, and 500 mg/mL of Arabica coffee husk extract can affect the hepatocytes of mice (*Mus Musculus*), so we use a dose one level above and one level below the effective dose to see the impact [14].

### 2.3. Inoculum Suspension

*P. gingivalis* ATCC 33277 (MediMark, France) was grown in TSA (Oxoid, England) containing 10% sheep blood, 0.4 μL/mL vitamin K1, and 5 μL/mL hemin and then grown for 21 days at 37°C in an anaerobic incubator. *P. gingivalis* for experimental periodontitis was determined to be 1.5 × 10^8^ CFU/mL equivalent to 0.5 McFarland standard.

### 2.4. *P. gingivalis* Injection

*P. gingivalis* bacterial suspension was injected into the gingival sulcus of the buccal part of the lower left first molar in the amount of 0.05 mL with a concentration of 0.5 McFarland (equivalent to 1.5 × 10^8^ CFU/mL). Injections are carried out for 21 days every 3 days to create chronic periodontitis conditions.

### 2.5. Tissue Preparation

The protocol applied for this animal experiment was approved by the Ethics Committee of the Faculty of Dentistry, Jember University, Jember. The rats were sacrificed at 21 days after the treatment. The left lower jaw was carried out using a scalpel. The specimens were then decalcified using 10% formic acid, embedded in paraffin, sectioned serially, and then stained with H&E to observe fibroblasts and Thricome Mallory to observe collagen density.

### 2.6. Statistical Analysis

The Statistical Package for the Social Sciences 26.0 software was used to analyze the data (SPSS for Windows; SPSS, Chicago, IL, USA). The acquired data were checked for normality with Shapiro–Wilk and homogeneity with Levene (*p* > 0.05: normal and homogeny data). The one-way ANOVA parametric test was employed for all treatment groups, followed by the post hoc LSD test to determine the significant differences in each group (*p* < 0.05: there are significant differences in each group).

## 3. Results

**Figure 1:**
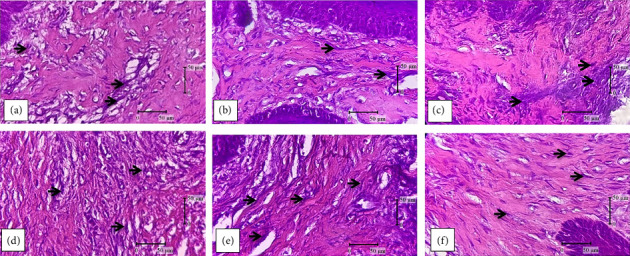
Black arrows indicate fibroblasts: (a) normal group; (b) negative control group; (c) positive control group; (d) robusta coffee husk extract group dose 125 mg/kgBW; (e) robusta coffee husk extract group dose 250 mg/kgBW; and (f) robusta coffee husk extract group with a dose of 500 mg/kgBW.

**Figure 2:**
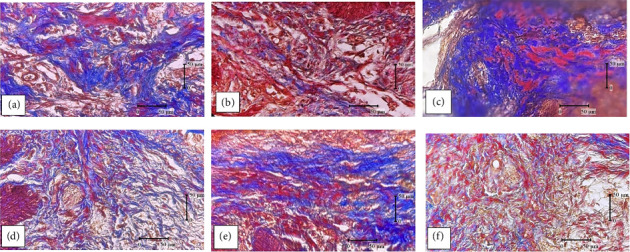
The blue fibers are collagen: (a) normal group; (b) negative control group; (c) positive control group; (d) robusta coffee husk extract group dose 125 mg/kgBW; (e) robusta coffee husk extract group dose 250 mg/kgBW; and (f) robusta coffee husk extract group with a dose of 500 mg/kgBW.

The results of research on Wistar rat gingival fibroblasts and collagen on Day 21 were observed using a light microscope with 400x magnification. Observation of fibroblasts was done using H&E staining (Figure 1). Collagen observation was done using Thricome Mallory staining (Figure 2).

**Figure 3:**
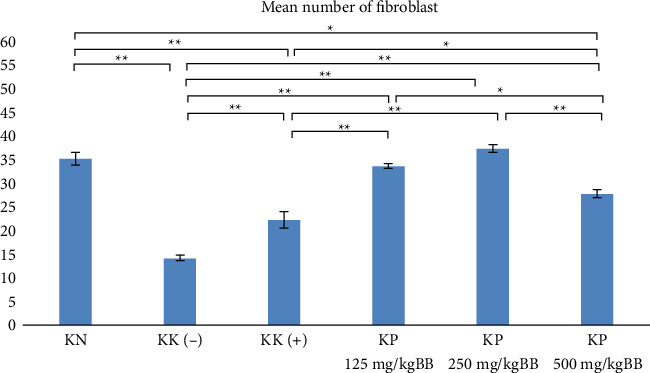
Mean number of fibroblasts (^∗^*p* < 0.05, ^∗∗^*p* < 0.01).

The results of calculating the number of fibroblasts showed that the highest average value was in the treatment group with a dose of 250 mg/kgBW (KP 250 mg/kgBW) while the lowest average value was in the negative control group (KK (−)). The treatment group had a higher average number of fibroblasts compared with the positive control group (KK (+)) that given metronidazole (Figure 3).

**Figure 4:**
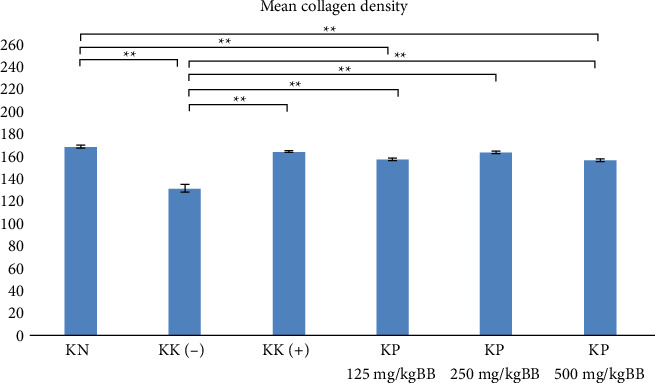
Mean collagen density (^∗^*p* < 0.05 and ^∗∗^*p* < 0.01).

Collagen density was observed using Adobe Photoshop software. The highest average collagen density was in the normal group (KN) while the lowest average was in KK (−). The treatment group with the robusta coffee fruit skin extract at a dose of 250 mg/kgBW had an average collagen density that was almost close to the average of the KK (+) given metronidazole (Figure 4). This increase is due to inflammation in the extract group which stimulates the tissue regeneration process, causing an increase in fibroblast proliferation to produce collagen. Increased number of fibroblasts results in higher collagen density. Increasing collagen production will increase the extracellular matrix so that tooth supporting tissue is formed more easily and facilitate periodontal tissue repair. Fibroblast and collagen are both crucial for the remodeling and homeostasis of periodontal tissue.

The Shapiro–Wilk test and Levene tests obtained results (*p* > 0.05), which showed that the data were normally distributed and homogeneous. The *p* value of the one-way ANOVA and post hoc LSD tests is 0.000, which means that there is a significant difference in the number of fibroblasts and collagen density between groups.

## 4. Discussion

Based on the results, the average number of fibroblasts and collagen density of gingival of rats in the extract treatment group with doses of 125, 250, and 500 mg/kgBW were greater than the KK (−). These results showed that giving robusta coffee husk extract to rats induced by *P. gingivalis* can increase the number of fibroblasts and collagen density. KK (−) had the smallest average number of fibroblasts and collagen density compared with all the study groups.

Due to the continuous induction of *P. gingivalis* without any treatment, thus causing inflammation to continue and damage tissue. LPS can stimulate macrophages to produce proinflammatory mediators, namely, TNF-α, IL-1, IL-6, and IL-1β and proteolytic enzymes such as MMP, especially MMP-8. MMP will degrade collagen fibers, causing damage to gingival connective tissue. Tissue damage will worsen in conditions of oxidative stress, namely, an imbalance in the production of reactive oxygen species (ROS) in cells and tissues [1, 15, 16]. Previous research shows that an excessive increase in ROS production can inhibit fibroblasts from forming an extracellular matrix, which results in the healing process being disrupted [17].

The treatment group with a dose of KP 250 mg/kgBW had the highest average number of fibroblasts and collagen density. This is because in the process of healing periodontitis, the main therapy is needed in the form of mechanical therapy (scaling and root planning) and also requires supporting therapy in the form of chemical therapy (antibiotics and anti-inflammatories) to prevent secondary infections and support the tissue healing process [18]. The flavonoid content in robusta coffee husk extract effectively inhibits immune cells from producing proinflammatory cytokines, thereby preventing inflammation from continuing [19]. The active compound that is also widely contained in robusta coffee husk extract is phenol. Other research shows that flavonoids and phenols can act as exogenous antioxidants that can stabilize ROS. Decreased amounts of ROS also help the fibroblast proliferation process by reducing cell death so that it can increase collagen production in the healing process [7, 20].

KK (+) had no significant difference in collagen density compared with the group given robusta coffee husk extract. These results show that treating periodontitis by administering metronidazole and robusta coffee husk extract can help the healing process because collagen density is found to be close to healthy tissue conditions. Others research shows that the flavonoid and phenol content in robusta coffee seed and husk extracts can act as an antibacterial against several bacteria, one of which is *P. gingivalis* [9, 21]. Previous research has shown that robusta coffee (*Coffea canephora*) husk extract at concentrations of 250, 500, 750, and 1000 mg/mL exhibited antibacterial activities against *P. gingivalis*. It was also found that the higher the concentration, the bigger the inhibition zone appeared [12]. Chlorogenic acid, which is one of the phenolic contents in robusta coffee husk extract, can damage cell structures so that bacteria will lyse [22]. The same healing results were also seen in the group given metronidazole. This is confirmed by previous research which stated that the use of metronidazole in cases of periodontitis showed a decrease in the number of bacteria, one of which was *P. Gingivalis* [23]. A decrease in the number of *P. gingivalis* can be interpreted as a reduction in bacteria that cause periodontitis. This can prevent continued inflammation thereby reducing collagen degradation [6].

Administering robusta coffee husk extract by sondage to mice aimed to ensure systemically effective delivery. Sondage allows the administration of the extract directly into the stomach, thus avoiding the digestive process that can reduce the effectiveness of bioactive substances. This ensures that the right dose of the extract can be optimally absorbed by the animal's body. With this method, the concentration of extracts in the systemic can be better controlled and can minimize fluctuations in the levels of active substances in the blood. Administering the extract through sondage can also help avoid gastrointestinal side effects that may occur if the animal tries to take the extract orally in liquid or solid form, such as nausea or vomiting [24, 25]. This administration process can increase the bioavailability of the bioactive components contained in the extract so that its therapeutic effect can be achieved faster and more efficiently to observe their effects on the number of fibroblasts and collagen density in gingival tissue.

The number of fibroblasts in the 250 mg/kgBW dose extract group on Day 21 showed an average higher than KN. This increase is due to inflammation in the extract group which stimulates the tissue regeneration process, causing an increase in fibroblast proliferation to produce collagen. Shafriani also stated that bitter gourd extract (*Trichosanthes cucumerina*) at a dose of 250 mg/kgBW can increase fibrinogen levels and reduce scars in diabetic ulcer model rats [26]. Increasing collagen production will increase the extracellular matrix so that healthy tooth supporting tissue is formed, especially gingiva. Clinically, healthy tooth supporting tissue is characterized by gingiva that feels supple, pink in color, no bleeding, the gingival margin is at the Cemento Enamel Junction (CEJ), etc. [27]. The research results obtained are in line with other studies which show an increase in fibroblasts starting on Day 3 and will continue to increase until Day 21 in the tissue regeneration process [11].

The 125 mg/kgBW treatment group had a higher average number of fibroblasts and collagen density compared with the 500 mg/kgBW treatment group. Therefore, it is necessary to carry out further measurements regarding the content of each substance in robusta coffee husk extract (phytochemical test). This is possible because the use of high doses of active substances can be toxic. Research by Cahyani et al. shows that the average liver cell necrosis is higher when flavonoids are administered at a dose of 500 mg/kgBW compared with a dose of 125 mg/kgBW, while a dose of 1000 mg/kgBW is toxic [14].

## 5. Conclusion

Based on the results of research that has been carried out, it can be concluded that robusta coffee husk extract at doses of 125, 250, and 500 mg/kgBW can increase the number of fibroblasts and collagen density in the gingiva of Wistar rats periodontitis. The most effective dose is the robusta coffee husk extract at a dose of 250 mg/kgBW. Robusta coffee husk extract can be an alternative periodontitis therapy and reduce the side effects of metronidazole as the main line of periodontitis treatment.

## Data Availability

The data and SAS code used to support the findings of this study are available on request from the corresponding author (Nadie Fatimatuzzahro: nadie.fkg@unej.ac.id).
